# A retrospective study of PBDEs and PCBs in human milk from the Faroe Islands

**DOI:** 10.1186/1476-069X-4-12

**Published:** 2005-07-14

**Authors:** Britta Fängström, Anna Strid, Philippe Grandjean, Pál Weihe, Åke Bergman

**Affiliations:** 1 Department of Environmental Chemistry, Stockholm University, SE-106 91 Stockholm, Sweden; 2 Institute of Public Health, University of Southern Denmark, DK-5000 Odense, Denmark; 3 Department of Environmental Health, Harvard School of Public Health, Boston, MA 02215, USA; 4 Faroese Hospital System, FR-100 Tórshavn, Faroe Islands

## Abstract

**Background:**

Persistent organic pollutants (POPs) in wildlife and humans remain a cause of global concern, both in regard to traditional POPs, such as the polychlorinated biphenyls (PCBs), and emerging POPs, such as the polybrominated diphenyl ethers (PBDEs). To determine the time related concentrations, we analyzed human milk for these substances at three time points between 1987 and 1999. Polychlorobiphenylols (OH-PCBs), the dominating class of PCB metabolites, some of which are known to be strongly retained in human blood, were also included in the assessment.

**Methods:**

We obtained milk from the Faroe Islands, where the population is exposed to POPs from their traditional diet (which may include pilot whale blubber). In addition to three pools, nine individual samples from the last time point were also analyzed. After cleanup, partitioning of neutral and acidic compounds, and separation of chemical classes, the analyses were carried out by gas chromatography and/or gas chromatography/mass spectrometry.

**Results:**

Compared to other European populations, the human milk had high PCB concentrations, with pool concentrations of 2300 ng/g fat 1987, 1600 ng/g fat in 1994, and 1800 ng/g fat in 1999 (based on the sum of eleven major PCB congeners). The nine individual samples showed great variation in PCB concentrations. The OH-PCBs were present in trace amounts only, at levels of approximately 1% of the PCB concentrations. The PBDE concentrations showed a clear increase over time, and their concentrations in human milk from 1999 are among the highest reported so far from Europe, with results of individual samples ranging from 4.7 to 13 ng/g fat

**Conclusion:**

Although remote from pollution sources, the Faroe Islands show high concentrations of POPs in human milk, particularly PCBs, but also PBDEs. The PBDEs show increasing concentrations over time. The OH-PCB metabolites are poorly transferred to human milk, which likely is related to their acidic character.

## Background

Even though persistent organic pollutants (POPs) have been regulated in most countries in the world, in agreement with the Stockholm convention in 2001 [1], the POPs are still posing a global environmental threat on the environment, both to wildlife and humans [2,3]. This is particularly true at hot spot locations and in remote areas where dietary habits may influence uptake and accumulation of POPs. This can be exemplified with human exposures to polychlorinated biphenyls (PCBs) at two former PCB production sites in Michalovche, Slovakia, and in Anniston, Alabama, U.S.A [4,5]. Populations with elevated levels of PCB due to food intake include populations frequently consuming fish from the Baltic Sea [6,7] and Lake Michigan, [8,9], Inuits from Northern Quebec [10,11] and Greenland [12], and the residents of the Faroe Islands [13,14]. The aim of the present study was to indicate any concentraion changes of two major classes of environmental contaminants in humans, as determined in human milk from the Faroe Islands, sampled at three different time points between 1987 and 1999. The chemicals chosen for analysis were PCBs and polybrominated diphenyl ethers (PBDEs), as well as polychlorobiphenylols (OH-PCBs) as the major class of PCB metabolites.

The Faroe Islands is a small community in the North Atlantic, located north of Scotland, far from industrial sources of any POPs, including both the PCBs and PBDEs. The Faroese population is exposed to these substances through their food, and possibly via goods and products in their homes and work environment. Their lifestyle is entirely Western and their diet is generally based on seafood. Part of the population has a considerable intake of traditional local food, such as pilot whale meat and blubber, and seabirds (e.g., fulmar meat and eggs) [13,15,16]. These high-trophic-level marine species are known to contain high concentrations of POPs (e.g. PCBs and PBDEs) [15-17]. The PBDE concentrations in pilot whale are in a concentration range similar to those reported in marine mammals from other parts of the world and considerably higher than those found in most marine mammals from the arctic [18,19]. Because of dietary differences among the Faroese, a wide variability in PCB concentrations is present. In 1987 milk pools contained 1.8–3.5 μg PCB per gram fat [20], serum from pregnant Faroese women in 1994 confirmed the overall average, but individual PCB concentrations ranged from 0.15 up to as much as 22 μg/g fat [13]. In subjects from the latter study, OH-PCB concentrations of 0.019 to 1.8 μg/g fat were reported, thereby showing the importance of this class of PCB metabolites retained in human blood [13].

PBDEs were first reported in fish from Sweden [21] and subsequently in Japan [22] and in human milk from Germany [23]. These initial results have been followed by a large number of studies world-wide; in humans, fish, birds and mammals [19,24]. In a time-trend study for PBDEs in human milk from Sweden from the early 1970s to 1997, the concentrations showed a significant increase [25], while samples from 1997 to 2000 indicate a decrease, mainly due to reduced concentrations of BDE-47 [26]. A decrease of PBDEs in Swedish milk was also indicated by Lind and co-workers 2003 [27]. A good overview of data on human PBDE exposures were given in recent reviews, including those by Hites [19], Sjödin *et al*. [28] and Ryan [29]. In Norway, PBDE in human milk increased from 1986 to 2001, with similar concentration levels to those reported in Sweden and Japan [25,26,30,31]. In the Canadian Artic, the PBDE concentrations have increased with a factor of 3 during the 1990s (i.e., from 2.2 to 6.2 ng/g fat) [32]. A similar concentration range has been reported from the United Kingdom. In southern Canada, the concentrations were about 3 times higher [29,32], and the PBDE levels reported in human milk from the United States are about 2–3 times higher again than those from Southern Canada and ~20 times higher than those seen in Europe and Japan [19,25,26,29,31,33,34].

PCBs still constitute some of the dominating environmental contaminants among all POPs [3]. The human concentrations of PCBs have decreased over time, even though the changes seem to be less than for 2,2-bis(4-chlorophenyl)-1,1-dichloroethene (DDE). These reductions in human PCB concentrations are related to the legislative measures taken in the 1970s. Temporal studies on the decrease of PCB levels, as studied in Swedish mothers milk from the early 1970s through 1997, indicate a 30% decrease [35]. In Germany, the PCB concentrations in mother's milk have decreased by 60% from 1986 to 1997 [36]. Also in Germany, Fürst and co-workers showed PCB decreases of up to 85% from 1984 to 2003 [37] (pers. comm. P. Fürst). Declining PCB concentrations in human milk have also been observed in Norway [38] and Canada [39], with different rates of the decrease in each country.

## Materials and methods

### Samples

In connection with formation of mother-child pair cohorts in 1987, 1994/95, and 1999 from consecutive births at the National Hospital in Tórshavn, Faroe Islands, milk was collected on days 3–5 post partum and then deep frozen. For the present study, mothers were selected from the three cohorts using the following inclusion criteria: age between 20 and 29 years, parity no more than one, singleton birth, and delivery at term. Similar procedures were used in 1987, 1994, and 1999 [20,40]. Three pooled milk samples were generated from the three cohorts, each containing the same amount of milk from ten mothers (2 ml from each mother). Because dietary habits are changing, women were further selected for inclusion in the study from information on their diet during pregnancy. The pooled samples contained milk from four women who did not eat whale meat at all during pregnancy, and two each who ate whale meat once, 2–3 times, and at least 4 times per month. However, due to lack of sufficient sample volume, the pool from 1994 contained milk from only three women who had not eaten whale meat at all. To determine the full range and variability of POP exposure, nine individual samples taken from the same women selected in the pool from the most recent cohort (1999) were also analysed. Due to limited volumes of milk available, specimens of at least 10 ml were taken from the milk samples collected 2–3 weeks after delivery. Additionally, due to changes in dietary habits in the Faroese [14], the samples selected from 1999 may not be fully representative of Faroese pregnant women, but they are comparable to the previously collected samples, in regard to traditional dietary habits. All samples were analyzed for major PBDE, PCB and OH-PCB congeners.

### Chemicals

The individual PBDE congeners (numbered according to Ballschmiter *et al*. [41]): BDE-47, BDE-77, BDE-99, BDE-100, BDE-153, BDE-154, and BDE-209 were synthesized in-house [42]. The individual PCB congeners: CB-101, CB-105, CB-118, CB-128, CB-138, CB-146, CB-153, CB-156, CB-170, CB-180, CB-183 and CB-200 [41] were purchased from Larodan Fine chemicals AB in Malmö, Sweden. The hydroxylated PCB standards 4-OH-CB146, 4-OH-CB187 and 4-OH-CB193 were synthesized (as described elsewhere [43]) and abbreviated according to Letcher *et al*. [44]. All solvents were of *pro analysis *quality. 2-Propanol from AnalaR (BDH laboratory supplies pool, England) and methyl *tert*-butyl ether (HPLC-grade; Rathburn, Walkerburn, Scotland) were glass-distilled prior to use. Silica gel (<0.063 mm) was purchased from Merck (Darmstadt, Germany) and activated (300°C, 12 h) before use.

### Instruments

The PBDE analysis was performed by gas chromatography/mass spectrometry (GC/MS) using a Finnigan TSQ 700 instrument (ThermoFinnigan, Bremen, Germany) connected to a Varian 3400 gas chromatograph equipped with an AS200S CTC autosampler. The transfer line temperature was set to 290°C and the ion source temperature maintained at 200°C. On-column injections were made using a septum-equipped temperature programmable injector (SPI) fitted with a high performance insert directly connected to a DB-5 HT capillary column (15 m × 0.25 mm i.d., 0.1 μm film thickness; J&W Scientific) with helium as carrier gas at a head pressure of 3 psi. The injector was temperature programmed from 60°C to 320°C at 150°C/min and the oven from 80°C (1 min), 15°C/min to 300°C (16 min). The PBDE congeners were analysed with selected ion monitoring (SIM) by scanning for the negative bromide ion (isotopes m/z 79 and 81) formed by electron capture reactions at chemical ionization (ECNI) with methane (5.0, AGA, Stockholm, Sweden) as the electron thermalization buffer gas at 5.6 torr and a primary electron energy of 70 eV. All chromatographic data were collected, analysed and quantified using the proprietary ICIS2 software from Thermofinnigan.

The PCB and OH-PCB analyses were performed on a Varian 3400 gas chromatograph, equipped with a Varian 8200 autosampler, an electron capture detector (ECD), and a split-splitless injector operated in the splitless mode. Hydrogen was used as a carrier gas and nitrogen as a make-up gas. A CP-Sil-8-column (25 m × 0.15 mm internal diameter and 0.12 μm film thickness Chrompack, EA Middleburg, The Netherlands) was used. For the PCB analysis, the column temperature was 80°C (1 min), 20°C/min, to 300°C (5 min) and for the OH-PCB analysis the column temperature was 80°C (1 min), 50°C/min, to 200°C (1 min), 1°C/min, to 230°C, 50°C/min, to 330°C (2 min). The injector temperature was 280°C and the detector temperature 360°C. The data were collected using a PC-based ELDS Pro v2.0 system (Chromatograhic Data System AB, Stockholm, Sweden). The linear relationship of the GC/ECD and GC/MS system was determined and the quantifications were performed using a single point external standard within the concentration range of the linear relationship.

### Extraction and cleanup procedure

The extraction and cleanup procedure for the milk samples is a modified version of a method for serum samples first described and validated by Hovander and co-workers [45]. In the modified version of the method, formic acid and diethyl ether are used instead of hydrochloric acid and methyl *tert*-butyl ether. Surrogate standards, BDE-77, CB-200 and 4-OH-CB193 were added to the samples prior to extraction. In short, formic acid (1 ml) and 2-propanol (6 ml) were added to a milk sample (5 g), subsequently extracted with a mixture of n-hexane/diethyl ether (1:1, 6 ml), and re-extracted once (3 ml). The lipid content was determined gravimetrically after gentle evaporation of the solvent. The neutral and phenol-type compounds were separated by partitioning with a potassium hydroxide solution. The bulk of lipids in the neutral fraction was removed with concentrated sulfuric acid and additional cleanup was performed on two subsequently applied sulfuric acid/silica gel columns, according to Hovander *et al*. [45]. Potential OH-PCB congeners were derivatized with diazomethane and any remaining lipids in the methylated phenol fraction were removed as described elsewhere [45]. All samples were protected from daylight during handling and storage to prevent any photochemical degradation of the brominated compounds to be analyzed.

Solvent blank samples representing every fifth sample were cleaned up and analyzed in the same way as the other samples. The PBDEs in the sample had to be three times greater than the PBDE amount in the blank to be considered as a quantifiable amount. The average blank sample amount has been subtracted from the results. The overall recoveries and standard deviation (SD) of the surrogate standards were 83% ± 6.1 for BDE-77, 83% ± 4.2 for CB-200 and 104% ± 6.7 for 4-OH-CB193.

### Recovery experiment

The recovery study was performed on 5 g of cow milk with a fat content at 3%. Before extraction a selected number of PBDE congeners were added, BDE-47, BDE-99, BDE-100, BDE-153, BDE-154 and BDE-209 at two different spike levels (0.1 ng/sample and 1 ng/sample) both in triplicates. In parallel, six samples were extracted without any PBDE congeners added. Prior to analysis by GC/MS, the same PBDE congeners (BDE-47, BDE-99, BDE-100, BDE-153, BDE-154 and BDE-209) were added to the samples at the two levels (0.1 ng/sample and 1 ng/sample). These samples therefore represent 100% of the analytes, and from these, the absolute recoveries were calculated. In addition, three more samples were run in parallel as blank samples for control of any background contamination.

## Results

**Figure 1:**
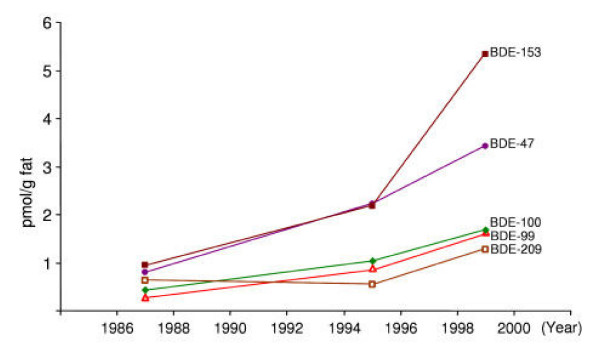
PBDE concentrations in pooled milk samples from the Faroese generated in 1987, 1994/95 and 1999. PBDE congener (BDE-47, BDE-99, BDE-100, BDE-153 and BDE-209) concentrations (pmol/g fat) in three pooled milk samples from mother-child pair cohorts generated in 1987, 1994/95 and 1999. Each pool consisted of 10 mothers and two samples from the same pool were analyzed in parallel.

**Figure 2:**
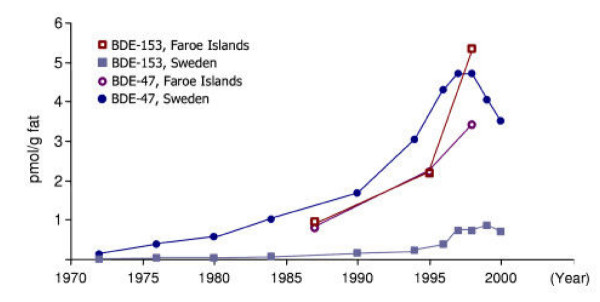
A time related comparison of BDE-47 and BDE-153 in Swedish mother's milk and the present study. Three pooled milk samples from mother-child cohorts generated in 1987, 1994/95 and 1999 from the Faroe Islands are compared to the Swedish pooled milk samples [25,26].

The duplicate results (A/B) for PBDE congener concentrations in pooled milk samples from 1987, 1994/95 and 1999 are presented in Table 1, as are the mean/medians and ranges for the nine individual samples from 1999. All PBDE results except for one were above the limit of quantification (LOQ), a single individual milk sample having a non-detectable BDE-209 concentration. The PBDE results for all individual samples from 1999 exceed the pool value from 1987, except for BDE-209 and BDE-154/BB-153. Likewise, all total PBDE concentrations of the individual samples exceed the totals from both 1987 and 1994/95. The time related levels of PBDEs (Fig. 1) therefore seems to reflect a real and substantial increase. While this is obvious for BDE-47, an even more dramatic increase has occurred for BDE-153, especially when compared to relevant data from Sweden (Fig. 2).

**Table 1:** Concentrations of the PBDE congeners identified in human milk from the Faroe Islands. The samples were pooled with ten mothers in each pool, two samples (A/B) of the same pool were analyzed in parallel. ΣPBDE consist of BDE-47, BDE-99, BDE-100, BDE-153 and BDE-209.

	Concentration (ng/g fat)				Concentration (pmol/g fat)			
Year	1987	1994/95	1999	1999	1987	1994/95	1999	1999
Samples	(A/B)^a^	(A/B)^a^	(A/B)^a^	n = 9^b^	(A/B)^a^	(A/B)^a^	(A/B)^a^	n = 9^b^
				mean/median (range)				mean/median (range)
BDE-47	0.37/0.40	1.1/1.1	1.7/1.6	1.9/1.3 (0.90–4.5)	0.77/0.83	2.2/2.2	3.5/3.4	4.0/2.6 (1.8–9.2)
BDE-99	0.15/0.17	0.50/0.51	0.91/0.94	0.84/0.73 (0.33–1.8)	0.26/0.30	0.88/0.90	1.6/1.7	1.5/1.3 (0.59–3.3)
BDE-100	0.23/0.26	0.58/0.59	0.92/0.96	1.0/0.58 (0.30–2.8)	0.41/0.45	1.0/1.1	1.6/1.7	1.8/1.0 (0.53–4.9)
BDE-153	0.57/0.65	1.4/1.4	3.3/3.6	2.4/2.1 (1.5–3.8)	0.89/1.0	2.1/2.2	5.1/5.5	3.8/3.2 (2.3–6.0)
BDE-209	0.59/0.59	0.47/0.55	1.1/1.3	1.0/0.60 (<0.14^c^–3.2)	0.62/0.62	0.49/0.58	1.2/1.3	1.1/0.63 (<0.15^c^–3.4)
ΣPBDE	1.9/2.1	4.0/4.2	8.0/8.4	7.2/5.8 (4.7–13)	2.9/3.2	6.8/6.9	13/14	12/8.9 (7.9–23)
BDE-154/BB-153	1.8/2.1	1.8/1.8	2.5/2.6	1.9/1.3 (0.66–4.0)	2.9/3.3	2.7/2.8	3.9/4.0	3.0/2.1 (1.0–6.3)

^a^The samples were pooled with 10 mothers in each pool, two samples of the same pool were analyzed in parallel.

^b^The nine individual samples are the same samples as those included in the pool, except one missing.

^c^LOQ for BDE-209

**Figure 3:**
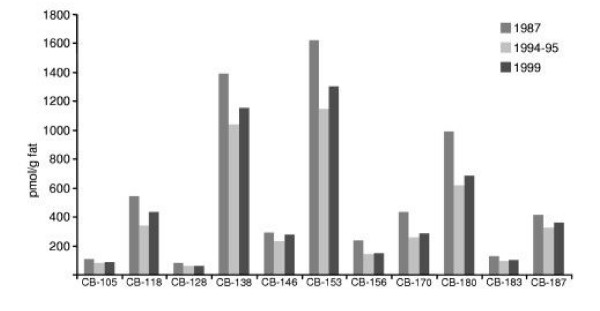
PCB concentrations in pooled milk samples from the Faroese generated in 1987, 1994/95 and 1999. PCB congener concentrations in pmol/g fat (CB-105, CB-118, CB-128, CB-138, CB-146, CB-153, CB-156, CB-170, CB-180, CB-183 and CB-187) in three pooled milk samples from a mother-child pair cohorts generated in 1987, 1994/95 and 1999. Each pool consisted of 10 mothers and two samples from the same pool were analyzed in parallel.

The concentrations of eleven major PCB congeners in pooled milk samples and the median and ranges for the nine individual samples from 1999 are given in Table 2. All concentration data are presented both on a weight and molar basis to promote correct comparisons of concentrations of these compounds with their highly different molar masses. In contrast to the tendency seen in PBDE concentrations, the PCB results are rather stable (Fig. 3), with the three pools showing quite similar results, and all pool data well within the variability seen in the individual samples from 1999.

**Table 2:** Concentrations of the PCB and OH-PCB congeners identified in human milk from the Faroe Islands. The samples were pooled with 10 mothers in each pool, duplicate samples (A/B) were analyzed in parallel. ΣPCB consists of CB-105, CB-118, CB-128, CB-138, CB-146, CB-153, CB-156, CB-170, CB- 180, CB-183 and CB-187 and ∑OH-PCB is the sum of 4-OH-CB146 and 4-OH-CB187.

	Concentration (ng/g fat)				Concentration (pmol/g fat)			
Year	1987	1994/95	1999	1999	1987	1994/95	1999	1999
Samples	(A/B)^a^	(A/B)^a^	(A/B)^a^	n = 9^b^	(A/B)^a^	(A/B)^a^	(A/B)^a^	n = 9^b^
				mean/median (range)				mean/median (range)
CB-105	32/39	28/27	27/32	31/19 (9.6–89)	98/120	85/83	83/98	95/57 (29–270)
CB-118	170/180	110/110	140/140	150/96 (42–430)	520/560	350/330	430/430	460/300 (130–1300)
CB-128/CB-167	30/31	21/21	22/23	22/17 (8.0–43)	82/85	58/59	62/63	60/48 (22–120)
CB-138	490/510	380/370	420/420	430/350 (160–1100)	1400/1400	1000/1000	1200/1200	1200/970 (450–3100)
CB-146	110/100	83/84	100/100	150/130 (55–450)	290/290	230/230	280/280	420/350 (150–1300)
CB-153	570/610	420/410	470/470	460/380 (180–1100)	1600/1700	1200/1100	1300/1300	1300/1100 (500–3000)
CB-156	85/88	53/50	56/55	52/42 (21–120)	230/240	150/140	150/150	140/120 (59–340)
CB-170	170/170	100/100	110/110	100/80 (41–230)	430/440	270/250	290/290	260/200 (100–570)
CB-180	380/400	250/240	270/270	250/210 (99–550)	970/1000	620/610	690/680	640/530 (250–1400)
CB-183	50/53	38/37	41/41	37/31 (14–88)	130/130	95/94	100/100	95/78 (36–220)
CB-187	160/170	130/130	140/140	130/110 (56–340)	410/420	330/330	360/360	330/270 (140–860)
ΣPCB	2200/2400	1600/1600	1800/1800	1800/1500(690–4600)	6100/6400	4400/4300	4900/4900	5000/4000 (1900–12000)
4-OH-CB146	0.80/0.87	0.33/0.26	0.35/0.40	0.66/0.36 (0.18–1.7)	2.1/2.3	0.88/0.69	0.93/1.1	1.7/0.96 (0.50–4.4)
4-OH-CB187	1.2/1.3	0.48/0.44	0.50/0.45	0.80/0.42 (0.26–1.8)	3.0/3.1	1.2/1.1	1.2/1.1	2.0/1.0 (0.64–4.5)
ΣOH-PCB	2.0/2.1	0.81/0.70	0.85/0.85	1.4/0.75 (0.45–3.5)	5.1/5.4	2.1/1.8	2.1/2.2	3.7/2.0 (1.1–8.9)

^a^The samples were pooled with 10 mothers in each pool, two samples of the same pool were analyzed in parallel

^b^The nine individual samples are the same samples as those included in the pool, except one missing.

Concentrations of two individual OH-PCB congeners (4-OH-CB146 and 4-OH-CB187) detected for all samples analyzed are presented in Table 2 (bottom). The ratio between the dominating OH-PCB metabolite and the PCB congener present in the highest concentration, 4-OH-CB187/CB-153 in the milk was below 0.002 in all cases.

The recoveries of BDE-47, BDE-99, BDE-100, BDE-153, BDE-154 and BDE-209 (Table 3) were about 90%, within a range of 79–107%, except for BDE-209 for which the recovery range was somewhat wider (86–160%). As an additional reflection of the analytical quality, the mean concentrations of the nine individual samples are very close to the value obtained from the pool, despite the fact that one of the single samples was lost. Although the individual samples were selected to represent differences in recent diets, there was no clear association between recent diet and the POP results obtained.

**Table 3:** Results from the PBDE recovery study performed in milk. PBDEs were added at two concentration levels.

	Low level ^a^	High level ^b^
	0.1 ng/sample	1 ng/sample

BDE-47	79 – 82	84 – 86
BDE-99	98 – 100	88 – 88
BDE-100	88 – 90	87 – 88
BDE-153	104 – 107	86 – 94
BDE-154	103 – 107	87 – 92
BDE-209	116 – 160^c^	86 – 105

^a ^Triplicates samples ^b ^Double samples ^c ^Only double samples

## Discussion

The concentrations of each of the PBDE congeners appear to increase substantially in human milk from the Faroese women studied between 1987 and 1999 (Table 1 and Fig. 1), most likely independent of a skewed sampling strategy. The variability of the ΣPBDE concentrations is apparent from analyses of the nine individual samples from the most recent group of samples (1999). The rather wide range between the highest and the lowest ΣPBDE concentration could conceivably be due to differences in life style and dietary habits, as has previously been demonstrated for PCB concentrations. Other studies have also revealed high variations in PBDE concentrations between individuals [27,34,46,47]. Previous studies have also shown increasing PBDE concentrations with time. For example, Merionyté and co-workers [25,26] showed a similar trend for BDE-47, but a very different trend for BDE-153 (Fig. 2). The apparent high concentrations and/or increase over time of BDE-153 in the Faroese is more pronounced than reported in most previous studies [28,31,34] even though this congener has occasionally been shown to dominate among the PBDE congeners in blood from humans in the Netherlands [47] and the human milk from the U.S. [48]. Very recently BDE-153 was pointed out as the most common PBDE congener in a few human adipose tissue samples [49]. The pattern with a high concentration of BDE-153 was seen in seven out of nine of the individual milk samples from 1999. The other PBDE congeners were present at concentration levels as expected from previously published data.

BDE-209 is a PBDE congener with an apparent short half-life (15 days) [50], and its presence in concentrations up to 3.2 ng/g fat, with only one sample below LOQ (Table 1) is an indication of continuous exposure to this PBDE congener. The median concentration of BDE-209 in 1999 is approximately half the concentration in serum from Swedish abattoir workers (2.5 ng/g fat) from 2000 [51]. BDE-209 was also recently detected at very low concentrations in some human milk samples from Germany collected in 2001–2003, with a median at 0.1 ng/g fat [52]. A mean of 0.9 ng/g fat was found in U.S. mothers [34], but only seven samples out of 47 were above the LOQ. In the Faroese milk samples, BDE-209 was above LOQ in all but one sample and at a mean concentration of approx. 1 ng/g fat at the last time point 1999. This is twice the concentrations of the milk pools from 1987 and 1994/95 (Table 1).

The ΣPBDE median concentrations of the nine individual samples and the level of the pool sample of the Faroe Islands mother's milk from 1999 are in the same range as milk samples from the United Kingdom mothers sampled in 2002 [33] and German samples from 2002 [53], but higher than those reported by Fürst and co-workers from southern Germany [37]. The Faroese human milk PBDE levels are intermediate between European and North American concentrations, with the exceptions mentioned [34]. Even though it may be convenient to compare overall ΣPBDE concentrations, individual PBDE congener concentrations (Fig. 1) are much more important, because the compounds differ in their toxicities. Depending on the large molecular weight span of the PBDE congeners, and given the high atomic weight of bromine, the PBDE congener concentrations are most correctly reported on a molar basis and accordingly comparisons are most correctly done this way.

The PCB concentrations (Table 2) in the Faroese human milk are considerably higher than those reported from other studies of human milk (e.g., in Sweden and Belgium), which are exceeded by a factor of about five in the milk from the Faroe Islands [35,54]. The PCB time trend observed in Swedish human milk showed a steady decrease, with the PCB concentration in 1997 reaching about 30% of that in 1972 [35]. In Germany, a decline to 10–25% of the PCB concentration occurred from 1984 and 2003 (P. Fürst, pers. comm.). In an Inuit population (Nunavik, Québec, Canada), the PCB concentration in cord blood were shown to decrease by about 7% per year from 1994 to 2001 [11]. All of these studies indicate decreases of PCBs in contrast to the relatively constant concentrations found in the present study among women in the Faroe Islands. However, a wide variation in the ΣPCB concentration occurs between the individual samples from the most recent group of the Faroese samples, and a small average decrease cannot be excluded. The relatively high concentration of CB-118 is notable, although the human milk PCB profile is dominated by CB-138, -153 and -180 (Table 2 and Fig. 3). Additional analyses would be necessary to describe the temporal trend of PCBs in human samples in the Faroe Islands.

The OH-PCB concentrations are low in mother's milk (Table 2). This finding is in accordance with results previously reported from Sweden and in Canada [55,56]. Hence 4-OH-CB187 was the dominating congener, followed by 4-OH-CB146 in the Faroese milk. The low concentrations are also to be anticipated, because these metabolites are mainly protein bound in the blood [44] and not dissolved or partitioned to lipids as are their parent compounds. Accordingly, the OH-PCB exposure of a nursing child is very low, despite the toxicant being easily transferred to the fetus [55,57]. These characteristics make the OH-PCB different from the neutral PCBs and PBDEs.

Despite substantial decreases elsewhere, Faroese human milk still contains high concentrations of PCBs, with levels in the low ppm range. The major PCB congener in human milk (CB-153) exceeds the most abundant PBDE congener (BDE-153) by a factor of 150 in the most recent samples (Tables 1 and 2). These high PCB concentrations are likely explained by dietary habits, given that the Faroe Island population has a high seafood intake, which includes PCB and PBDE contaminated pilot whale blubber, fulmars and fulmar eggs [15-17].

The high concentrations and unique pattern of PBDEs, with BDE-153 being the dominant congener, could possibly be explained by a higher persistence of BDE-153 than of BDE-47. In contrast, pilot whale blubber has been shown to contain mainly BDE-47, followed by BDE-99, then BDE-153, thus indicating that this food item may not be the primary source of human PBDE exposure. Since fulmars contain only low PBDE concentrations, these birds also constitute only a minor source for human PBDE intake. The high PBDE concentrations in 1999 are unlikely to be explained by exposures to electronic and electric equipments, since such exposures would not exceed those present in the rest of Europe. It is possible that at least some PBDEs are passed through marine food chains, and that the Faroese are highly exposed from their traditional seafood diet.

A study from a Northern Canadian population showed that the traditional food sources in this area did not necessarily result in higher concentrations of PBDEs, as observed for other persistent organic pollutants (POPs), such as the PCBs [58]. Arctic mammals, including ringed seals, have shown relatively low levels of PBDEs, ranging from 0.40 to 4.3 ng/g fat, as compared to pilot whale blubber from the Faroe Islands, where PBDE concentrations were 0.84 to 3.2 μg/g fat [17,18]. Still, the fact remains that residents at the Faroe Islands have high concentrations of both PCB and PBDE, and the difficulty in explaining the sources of the PBDEs suggest that this issue needs further attention.

The method used for the PBDE analysis in human milk has previously been used for analysis of PCBs in human milk and was formerly used also for both organochlorine and organobrominated compounds in human serum. The human serum method was adopted for human milk analysis with a few modifications [45]. The recovery study was performed with cow's milk [59], because the PBDE concentrations in human milk are much higher and would not be suitable for blank samples. A small pilot study was performed to confirm that the cow's milk was virtually free from any PBDEs, which was the case. Milk with 3% fat was used, because human milk normally has a lipid concentration of about that level. The recovery study was performed only for the PBDEs, because the analytical method for PCBs has been previously documented [60]. The average recoveries of BDE-47, BDE-99, BDE-100, BDE-153 and BDE-154 added to the cow milk before extraction ranged from 79 – 107% (Table 3).

## Conclusion

This study reports PBDE concentrations in human milk from the Faroe Islands, for the first time. A steep increase of PBDE concentrations is shown from 1987 to 1999, and the concentrations in milk from the late 1990s are among the highest in Europe. The PBDE pattern is different from the one reported elsewhere, with BDE-153 as the dominant congener, rather than BDE-47 [25,30]. The PBDE sources for the Faroese population still need to be identified, but could include food items affected by passage through marine food chains. Furthermore, the PCB concentrations remain high in Faroese mother's milk with no clear decrease over time up to the late 1990s. The hydroxylated metabolites of PCBs are poorly transferred to mother's milk leading to very low exposures to these metabolites via the milk.

## List of abbreviations

GC/ECD gas chromatography / electron capture detector

GC/MS gas chromatography / mass spectrometry

OH-PCBs polychlorobiphenylols

PBDEs polybrominated diphenyl ethers

PCBs polychlorinated biphenyls

POPs persistent organic pollutants

## Competing interests

The author(s) declare that they have no competing interests.

## Authors' contributions

BF led the writing of the manuscript, assisted in the clean-up procedure, the GC/ECD and GC/MS analysis of the samples, and designed the project. AS did the cleanup work for the samples and made the GC/ECD analysis. PG participated in the writing and design of the project. PW collected and selected all the samples. ÅB participated in planning, writing, and interpretation. All authors read and approved the final manuscript.
